# Corneal Behavior during Tonometer Measurement during the Water Drinking Test in Eyes with XEN GelStent in Comparison to Non-Implanted Eyes

**DOI:** 10.3390/jcm11112962

**Published:** 2022-05-24

**Authors:** Agnieszka Jóźwik, Joanna Przeździecka-Dołyk, Ewa Wałek, Magdalena Czerniak, Magdalena Asejczyk

**Affiliations:** 1 Department of Optics and Photonics, Wrocław University of Science and Technology, Wybrzeże Wyspiańskiego 27, 50-370 Wrocław, Poland; agnieszka.jozwik@pwr.edu.pl (A.J.); 236813@pwr.edu.pl (M.C.); magdalena.asejczyk@pwr.edu.pl (M.A.); 2 Department of Ophthalmology, Wroclaw Medical University, Borowska 213, 50-556 Wrocław, Poland; ewka.walek@gmail.com

**Keywords:** XEN GelStent, corneal hysteresis, corneal resistance factor, open-angle glaucoma, intraocular pressure

## Abstract

Biomechanics of the cornea have significant influences on the non-contact measurement of the intraocular pressure. The corneal behaviour during tonometry is a fundamental factor in estimating its value. The aim of the study was to analyse the behaviour of the cornea during tonometric measurement with the forced change in intraocular pressure during the water drinking test. Ocular Response Analyser (Reichert) was used to the measurement. Besides four basic parameters connected with intraocular pressure (IOPg, IOPcc) and biomechanics (corneal hysteresis CH and corneal resistance factor (CRF), other parameters representing the behaviour of the cornea during a puff of air were analysed. There were 47 eyes included in the study, including 27 eyes with a XEN GelStent implanted and 20 without it. The eyes of people with monocular implementation were the reference group. The values of analysed parameters were compared before and after 10, 25, 40, and 55 min after drinking the water. The intraocular pressure increased by 2.4 mmHg (*p* < 0.05) for eyes with a XEN stent and 2.2 mmHg for eyes without a stent (*p* < 0.05) in the tenth minute after drinking of water. This change caused a decreasing of corneal hysteresis (*p* < 0.05) without significant changes in the corneal resistance factor (*p* > 0.05). Corneal hysteresis changed similarly in the reference group and the group with a XEN GelStent. The analysis of additional parameters showed a difference in the behaviour of the cornea in eyes with a XEN GelStent in comparison to the corneas of eyes without a stent. This was particularly visible in the analysis of the cornea’s behaviour during the second applanation, when the cornea returns to its baseline state after deformation caused by air puff tonometry.

## 1. Introduction

Assessment of eye biomechanics is crucial for the adequate understanding of corneal behaviour in response to mechanical actions in its structure, refractive surgery-induced tissue remodelling, or non-ablative refractive correction, as well as aspects of corneal physiology processes. Changes in its behaviour are the basic factor in estimating its value, especially during non-contact measurement. In 2001, David Luce showed that, from the signal received by the non-contact tonometer, it is possible to obtain information not only about the intraocular pressure but also about the biomechanical properties of the cornea [1]. Corneal hysteresis (CH) parameter, representing the viscoelastic nature of the cornea, became available by using a commercial device—Ocular Response Analyzer (ORA). It was shown, before, that both types of the intraocular pressure measured by ORA (Goldmann-correlated pressure IOPg and corneal-compensated pressure IOPcc) have good agreement with GAT on normal subjects [2]. IOPcc compared to GAT suggests IOPcc shows greater agreement with GAT than ORA IOPg [3].

Glaucoma, which is a disease defined primarily on the basis of changes in the optic nerve disc, also affects the biomechanics of the eyeball. One of the main factors influencing the development of glaucoma is the intraocular pressure increasing. It could cause changes in the structure of the eyeball, e.g., loss of corneal endothelial cells. This is important in primary angle closure glaucoma because, if a high IOP level remains elevated longer than 72 h, an irreversible, very large, and significant loss of endothelial cells may occur in the cornea [4]. Biomechanical parameters can be regarded as biomarkers of glaucoma susceptibility [5]. Corneal hysteresis CH in glaucomatous eyes is lower than in the healthy eyes, and this decrease is correlated with a reduction in the field of view [6]. However, the relationship between the biomechanics of the cornea and the appearance of the optic disc is still not fully known [5]. However, studies have shown that this parameter can be used to predict the progression of primary open-angle glaucoma [7,8,9,10]. The authors of the studies mentioned above reported that the progression of glaucoma is likely to be influenced by the biomechanical properties of the cornea. Researchers observed that CH measurements were significantly less in primary open-angle glaucoma and normal-tension glaucoma compared to normal subjects. Besides CH, the device gives information about corneal resistance factor (CRF). CRF is a measure of the overall resistance of the cornea related to its elastic properties. CRF is not associated with CH. Despite many researchers of this subject, there is no so clear understanding of how CRF is correlated with the occurrence of glaucoma. Some researchers show that CRF is not influenced by glaucoma [8]. However, there are results showing that CRF was significantly less in normal-tension glaucoma and maximum in primary open-angle glaucoma and ocular hypertension [9]. However, it was noted that procedures aimed at lowering the intraocular pressure, including glaucoma drainage, trabeculectomy, and trabeculectomy combined with cataract surgery, may lead to changes in CH and CRF parameters. Research conducted at the Ophthalmic Research Center in Tehran in 2014 showed that the values of CH and CRF parameters increase after successful IOP reduction and cataract removal procedures [11]. These studies analysed the results after trabeculectomy, phaco-trabeculectomy, Ahmed glaucoma valve implantation, and phacoemulsification. Refractive surgery procedures also affect the change in CH and CRF parameters, but the opposite relationship is observed compared to cases of glaucoma treatment. The values of the CH and CRF parameters decrease after these procedures [12].

One of the solutions to stop the progression of the glaucoma is the implantation of drainage microducts as a part of minimally invasive glaucoma surgery (MIGS). Such stents allow an additional path of the flow of aqueous fluid between the anterior chamber and the preconjunctival space. One of the MIGS devices is XEN GelStent (Allergan, Dublin, Ireland), which is a 6-mm long stent of collagen-derived gelatin cross-linked with glutaraldehyde. The procedure is almost always augmented with a subconjunctival or sub-Tenon injection of mitomycin C. The XEN GelStent received the CE mark approval in 2011 and the US FDA approval in 2016. The stent was inserted into the eye using a 0.4 mm diameter needle in the upper-nasal quadrant of the eyeball. The implant starts working as soon as it is placed in the eye [13]. The previous work [14] has shown if such an implementation has an impact on the biomechanics of the cornea. CRF and CH changed significantly in eyes with primary open-angle glaucoma after XEN GelStent implantation (post-XEN), in comparison to primary open-angle glaucoma control group (eyes without stent). Analysis of the basic parameters from ORA showed that the biomechanical parameters of the anterior chamber in the post-XEN group changed significantly during WDT (water drinking test).

The Ocular Response Analyzer is an air-puff tonometer. An impulse of air is released towards the cornea. It bends inward and, after reaching the maximum deflection, it returns to its initial state. During the movement both inwards and backwards, the cornea passes through the applanation state, which is equivalent to a flattening, corresponding to the one prevailing in the Goldman tonometry. Corneal deformation is recorded with an electro-optical infrared detection system, and results are presented as the applanation curve. The shape of the signal curve is obtained from ORA, and therefore, the parameters describing this curve may also reflect the biomechanical properties of the cornea, as well as the parameter CH [15]. Changes in the shape of this signal indicate various pathological deviations of the cornea, including keratoconus [16]. It was showed that the shape of this signal is also different after refractive surgery [17].

A symmetry of eyes was observed in individual patients, e.g., an astigmatic axis, IOP, higher-order aberrations, corneal curvature, and corneal thickness [18,19,20,21]. The symmetry of biomechanical parameters is purely investigated. However, symmetry of CH and CRF was proven [22,23].

The aim of the study is to investigate the influence the XEN GelStent drainage implementation on corneal biomechanical behaviour. This research is based on a comparison of the parameters, describing the deformation of the cornea, during air-puff measurement in the eyes with the stent in relation to the eyes without it.

## 2. Methods

The present study was registered in ClinicalTrials.gov with the number NCT03904381 and conducted at the Department of Ophthalmology, Wroclaw Medical University. Clinical data base were collected between April and May 2018. The screening phase lasts between June 2018 and August and measurements between September and December 2018. All patients were informed about the aim, benefits, and risks of all procedures of the study before screening phase. The study was performed in adherence to the Declaration of Helsinki and was approved by the local Ethics Committee (approval number: KB 563/2017).

### 2.1. Measurement Group

The study included 39 patients in the screening phase after XEN GelStent implantation. The 27 patients were observed in 12 months of follow-up period. Finally, 18 patients were enrolled in the research. All subjects had a XEN GelStent implant in one eye (XEN group). The other eyes without a stent were considered as a reference POAG group (Control Group). The mean age of patients was 69 ± 12 years (range: 34–81 years).

Patients with POAG after at least 3 months of post-XEN GelStent implantation were prospectively enrolled in the study between September and December 2018, and details concerning glaucoma stage, medication status, and further information are summarised in Supplementary Files–Table S1. Technical details of implantation and the perioperative period have been described previously [24]. Patients with a reduction in IOP, compared to the pre-XEN implantation measurements of at least ≥20% baseline IOP and ≤21 mmHg, as well as at least 3 months post last 5-FU injection, were included to the research. With any progression (according to the EGS guidelines) within the last 3 months, any change in medication within the last month, and any systemic medication within the last 3 or more months, 5-FU injections were used as exclusion criteria. A reference group consists of POAG patients with at least 3 months on stable local anti-glaucoma medications without significant side effects and a reduction in IOP compared to the pre-medication measurements of at least ≥20% baseline IOP and ≤21 mmHg. Any progression (according to the EGS guidelines) within the last 3 months, or any procedures using lasers, were criteria for excluding eyes from the research. High axial refractive errors, due to elongation of the globe (AXL > 26 mm), also excluded patients of both groups from the research. None of the included subjects took any systemic antiglaucomatous medication at least 3 months previously. Only three eyes (16%) in the XEN group required topical anti-glaucoma medications to control IOP (one patient was taking β-blocker, and two patients were taking prostaglandins eye drops). All participants underwent complete ophthalmological examination before participation to determine their refractive and health status. The detailed procedure was described previously [14].

### 2.2. Ocular Response Analyzer and Measurement Procedure

**Figure 1:**
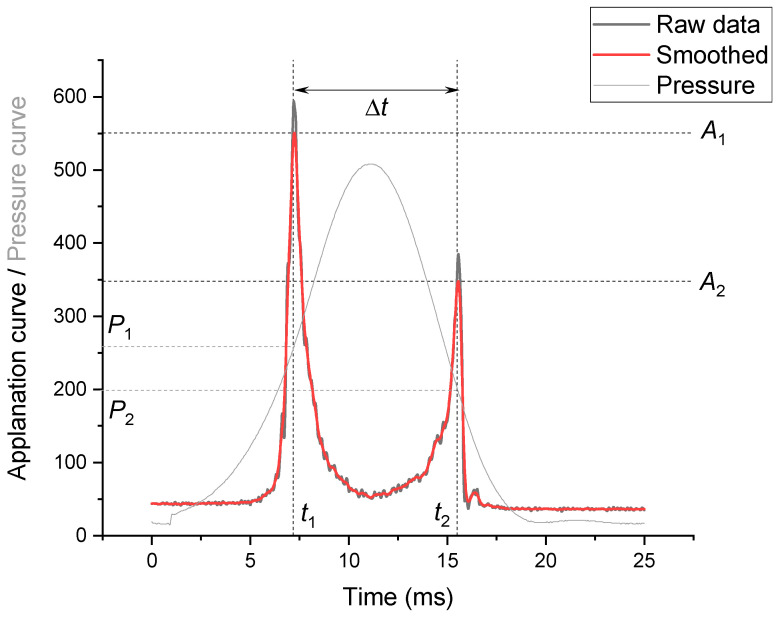
The applanation curve recorded by ORA (control group, before WDT, patient 11).

The biomechanical data were measured using ORA (Reichert Ophthalmic Instruments, Inc., Buffalo, NY, USA; Software version 3.0). Basically, ORA generates two separate parameters related to the intraocular pressure: Goldmann-correlated pressure (IOPg) and corneal-compensated pressure (IOPcc). In addition, using a bidirectional applanation measurement, ORA allows the determination of two parameters describing the biomechanical properties of the cornea: Corneal Hysteresis (CH) and Corneal Resistance Factor (CRF). The quality of measurement is described by the waveform score (WS) on a scale from 0 to 10, which indicates the reliability of each measurement. The deformation of the cornea is recorded with an electro-optical infrared detection system that records the intensity of light reflected from the surface of the cornea as it is deflected. There are 400 points of the light deflection that are recorded during a 25 ms period, and they form the applanation curve (black line). An exemplary applanation curve is presented in Figure 1. The applanation curve is characterized by the two characteristic peaks, which correspond to the moments of corneal flattening, when the pressure on both sides of the cornea is equalized. Additionally the air-pressure curve, representing pressure of the air flow emitted by the device, is recorded (Figure 1, grey line). Based on these two curves, the pressure during applanation states is determined. The pressure 𝑃1 is recorded for the first applanation, occurring as the cornea moves inward with an increasing air pulse, while 𝑃2 is the pressure corresponding to the second applanation when the cornea returns to its initial curvature while decreasing the stream of the air. Therefore, for corneas after intervention to its structure, the mechanical response and the entire applanation curve may be very irregular. In this case, the applanation peaks could be lower, wider, or otherwise irregular. 

The ORA additionally determines 37 parameters describing the shape of the applanation curve representing the change of the curvature of the cornea during the measurement. They represent properties of corneal behaviour separately in two applanation areas, including the area under the curve (p1area, p2area), upward slope (uslope1, uslope2), downward slope (dslope1, dslope2), peak width (w1, w2), peak height (h1, h2)), length of the curve around the peaks (path1, path2), smoothness of the peaks (aindex, bindex), noise (aplhf), and six additional parameters (dive1, dive2, mslew1, mselw2, slew1, and slew2). A detailed description of the parameters is presented in the Supplementary Files–Table S2.

Additionally, the amplitudes (*A*_1_ and *A*_2_) and the times (*t*_1_ and *t*_2_) of both applanation occurrences were calculated from raw data taken from the device (Figure 1). Firstly, the applanation curve was smoothed with Gaussian estimation and with the window size 11. Smoothing was supposed to minimize sharpness of the raw data curves (Figure 1, red line). Moreover, the difference between applanation times (Δ*t*) was calculated. 

The water drinking test was used to obtain different levels of the intraocular pressure. The patient drank an amount of the water proportional to his weight, with the proportion 10 mL/kg, for 10 min. The values of parameters measured by the ORA were recorded before (reference result) and 10, 25, 40, and 55 min after stopping drinking the water. The biomechanical properties were measured, fourfold, with a waveform score higher than 5, and mean values were recorded for further analysis. All recordings were conducted on the same equipment by the same dedicated examiner. After the conduction of WDT, each patient was observed for a year, and control visits were conducted as described in the study protocol (NCT03904381).

### 2.3. Statistical Analysis

Statistical analyses were conducted using Statistica Software version 13.3 (TIBCO Statistica 1984–2017 TIBCO Software Inc., Palo Alto, CA, USA)) licensed by Wroclaw University of Science and Technology. The results of measurements of 4 basic parameters (IOPg, IOPcc, CH, and CRF), 37 additional parameters describing the deformation of curves signals of eyes, and 5 parameters defined in this work, in the eyes after stent implantation in relation to eyes without an implant, were compared. The Shapiro–Wilk test was used to check the normality of the sample distribution. Repeated measures analysis of variance (ANOVA and Friedmann), with the Bonferroni or Dunn adjustment for multiple comparisons, was used to determine the influence of the within-subjects factor water unload. The nonparametric Wilcoxon rank-sum or paired *t*-test were used to evaluate the distribution of variables between the two groups (subject groups: post-XEN and control). Results were considered statistically significant with a *p* < 0.05.

## 3. Results

The study included the post-XEN group with 18 eyes and 18 non-implanted eyes (control group). The group had a similar female/male ratio (*p* > 0.05). No differences in the baseline GAT, central corneal radius CCT, ACD, AXL, BCVA, MD, PSD, RNFLT, body weight, BMI, ECC, and hexagonity of endothelial cells were recorded (Table 1). 

### 3.1. Intraocular Pressure during the WDT

According to the pre-specified criteria, WDT was positive. Results of the intraocular pressure are presented in Table 2. Before the water drinking test, the intraocular pressure of the eyes with XEN GelStent implants was lower by 2 mmHg, on average, than in the eyes in the control group. This trend continued at a similar level. However, these differences were not statistically significant. It is worth noting that values of IOPcc (corneal compensated pressure) were higher than IOPg (Goldmann-correlated pressure). It could be caused by thinner central corneal thickness, of the considered group (CCT about 530 μm), than average for healthy eyes. Independently of the group, the highest increase in intraocular pressure was observed 10 min after drinking of water. The increase after 10 min and 25 min was statistically significant (*p* < 0.05 post-hoc analysis). After this time, the IOP returned to the pre-test level in the group of eyes with the stent. This stabilisation was also observed in the control group, but it followed slower than in the XEN GelStent group. 

### 3.2. The Biomechanical Parameters during the WDT

During the analysis of the biomechanical parameters, it was observed that CH in the control group changed significantly during WDT (*p* < 0.05), but there were not changes in CRF (*p* = 0.17). The biomechanical parameters of the anterior chamber in the XEN GelStent presented no statistical changes in WDT (*p* = 0.17 and *p* = 0.41, for CH and CRF, respectively). In the XEN GelStent, there were no statistically significant changes for CH, but the variation for CH were observed during the measurement. The corneal hysteresis decreased, both in CG and XEN GelStent groups, during particular parts of measurement, while CRF shows a similar trend to the intraocular pressure, i.e., it increased after 10 min and then decreased. Detailed results are presented in Table 3. There was not a difference between groups on each level of the measurement during the water drinking test. 

### 3.3. Analysis of the Applanation Curve Parameters

**Figure 2:**
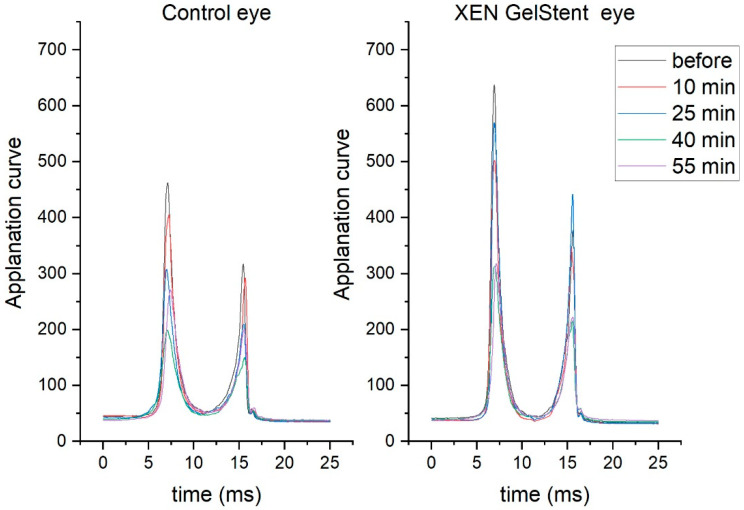
Exemplary applanation curves for one patient during the water drinking test.

Different behaviour of the cornea was observed in eyes with the XEN GelStent implant, in comparison to non-implanted eyes, the during the water drinking test. Exemplary applanation curves, for one patient during the water drinking test, are presented in the Figure 2. Among individuals, 50% had higher peaks in the first applanation (A1) in XEN GelStent eyes, and 38% had peaks in the second applanation (A2). Among individuals, 45% had symmetrical behaviour in both eyes, which means there was a decrease or an increase in the peak height in both eyes simultaneously. A decrease in the maximum value, related to the first applanation, was observed in 62% of both the control and XEN GelStent eyes, but it did not occur in the same pair of eyes. During the second applanation, the lower maximum was observed in 80% of the control eyes and 38% of the XEN GelStent eyes. 

The further analysis was divided into two parts related to the first and the second applanation. All results are present in Supplementary Files–Table S3, respectively. The only parameter that is not directly related to both applanations is aplhf, representing noise between applanations. Similarly, parameters aindex and bindex, which are correlated with the quality of the curves (during the first and the second applanation, respectively), are not susceptible to WDT. The analysis showed that changes caused by drinking water did not change the value of this parameter (Friedmann test, *p* > 0.05). Most of the analysed parameters were lower on each step of the measurement during the water drinking test. Some of them returned to the pre-WDT level after 55 min. In fact, only the width of the peaks (w1, w2, w21) behaved similarly to the tendency of the intraocular pressure or CRF. It means that there was an increase in the first 10 min after water drinking, and then, it declines, and this change was statistically significant for the control group only (*p* < 0.05).

Only a few of the analysed parameters did not change during WDT in both groups. During the first applanation, no change was observed for absolute value of path length around the peak (path1, path11) and the maximum single step increase in the rise of the peak (mslew1). During the second applanation, there were the parameters describing the area of the peak (p2area and p2area1).

Despite the lack of statistically significant differences in all analysed parameters for considered groups, some trends were observed in the parameters’ values. Regardless of the applanation type, higher peaks (h1, h11, h2, h21) were obtained in the control eyes group in comparison of XEN GelStent group. There were also thicker peaks during the first (w1, w11) and wider peaks during the second (w2, w21) applanation. Analysis of slopes showed slightly lower values of the parameters describing the first applanation (uslope1, uslope11, dslope1, dslope11) in XEN GelStent group than in the control group, but it showed higher values describing the second peak (uslope2, uslope21, dslope2, dslope21). A similar tendency had the aspect ratio of the peak, calculated as dividing height by width of the peak. The aspect ratio of peak 1 (aspect 1 and aspect 11) was higher for the control group, but for peak 2 (aspect 2 and aspect 21), it was higher for the XEN GelStent group. All these changes were small and statistically insignificant. 

### 3.4. Analysis of Parameters from Raw Data of Applanation Curve

The analysis of the maximum value of two peaks calculated from raw data is similar to previously presented h1, h11, h2, and h21 because their determination is of a similar nature (Table 4).

The times of applanation occurrence differentiate for the control and XEN GelStent groups, especially when considering the interspace between applanations (Δ*t*). The eyes with implants reacted faster to the air-puff stream than the control eyes, on particular stages of the measurement, but simultaneously when the second applanation is delayed. P-values are on the margin of the statistical significance for comparison of parameters *t*_1_ and Δ*t* between two groups of eyes (Table 5).

The influence of WDT is observed in the analysis of the first applanation time. The time of the second applanation did not change significantly during WDT.

## 4. Conclusions

Biomechanics of the cornea are important in many diagnostic procedures. It was interesting to check whether the placement of the implant disturbs such corneal biomechanics of the eye and could influence the results of the intraocular pressure measurements. The analysis was performed based on the deflection cornea during the air-puff tonometry. The ORA, one of the most commonly used tonometers, has become a popular clinical device for evaluating biomechanical properties. CH and CRF are parameters that are important factors in understanding the biomechanical state of the cornea and the clinical diagnosis of eye diseases. ORA gives the possibility to analyse additional parameters, describing the shape of the applanation curve and being a representation of the corneal deflection, during the air-puff measurement. 

The analysis is based on evaluating the behaviour of the cornea while implementing a water drinking test (WTD), with the aim being to check the influence of an increasing pressure on the biomechanical properties. The water drinking test was performed in two groups of eyes. One group included eyes with the XEN GelStent implant, and the control group consisted of the second eyes of the same patients (POAG eyes, usually with pharmacological treatment). Before WDT, there was a difference observed in the initial intraocular pressure. Higher values of IOP were observed in the control group of eyes. This means that the implant is working properly. The pressure increased 10 min after drinking water, by more than 2 mmHg, in both groups. A higher or lower increase in IOP depends, mainly, on the efficiency of the choroidal scleral outflow tract. Pressure returned to the pre-water level faster in eyes with the stent. It was especially seen in corneal compensated IOP (IOPcc). In earlier work [6,11,25], authors reported that CH parameters increase after IOP reduction. In this research, this increase is not statistically significant for comparison of the control and adequate XEN GelStent eyes. However, CH decreases after water drinking, and this change was statistically significant in the control eyes (*p* < 0.05). Subsequently, this value remained at a similar level. The lack of noticeable changes, compared to values before WDT, may be due to corneal hydration. In the XEN GelStent group, CRF was lower than in the control group, but this difference was not significant. The value of the CRF parameter did not change as a consequence of drinking water, so it can be established that the corneal resistance is a parameter that is more stable and can describe the biomechanics of the cornea more precisely. Observed higher CRF and lower CH, in 10 min after water drinking, could be indicative of a “protective effect” associated with a greater central corneal thickness in higher pressure eyes, where more force was required to induce applanation. Therefore, one would expect an overestimation of the IOP in a cornea with a higher CRF [9]. Sharifipour et al., also observed that CH and CRF changed significantly after WDT in medically or surgically (trabeculectomy) controlled glaucoma in comparison to normal individuals [26]. In this research, while CH changes after WDT were not significantly different among the groups, CRF changes in the medical group were significantly higher than the control group. 

Basic parameters from Ocular Response Analyser are calculated from pressures obtained during two applanation states when the cornea deflects inwards (P1) and towards (P2). The calculated pressure depends on the time of the applanation. Our research was showing that the first applanation (*t*_1_) was observed about 0.2–0.3 ms later, for the control, in comparison with the XEN GelStent group. It was expected since the cornea in eyes with higher intraocular pressure are more resistant to deflection. It also takes longer for the cornea in XEN GelStent eyes to return to its original shape. The second applanation (*t_2_*) was achieved slightly later for XEN GelStent eyes, but this delay was not statistically significant. The time of the second applanation (*t*_2_) did not change significantly during WDT in contrast to the time *t*_1_. Based on this, it can be concluded that the second peak of the applanation curve may better reflect the corneal biomechanics, and the first peak is more related to the intraocular pressure. The most important observation is related to Δ*t* before drinking water and shortly after there were statistically significant differences in the intervals between the applanations. After 25 min, these intervals did not differ significantly. This time was longer for the eyes with the implant.

Changes in the shape of the curves describing the intensity of light reflected from the cornea were also observed, but these differences were not statistically significant between both eyes. An analysis showed that both peaks for control eyes were higher than for XEN GelStent group, wherein the first peak was narrower, and the second peak was wider for XEN GelStent eyes. It means that the applanation area was wider for control eyes. The parameters describing the slope of the peaks, representing the rate of achieving applanation, showed that, in the XEN GelStent group, the first applanation was slower than in the control group. However, in the case of the second application, it was the other way around, and for the eyes with XEN GelStent, these speeds were higher. It could mean a slight stiffening of the cornea in eyes with the implant, or it may be caused by a change in the biomechanical properties, such as viscoelasticity of the cornea, by the stent implantation procedure. This conclusion is controversial in literature, although recent research showed that this procedure did not have to have a direct effect on the corneal endothelial cells and, thus, on its structure [27].

Most analysed parameters were sensitive to the influence of WDT in both groups. Almost all value parameters decreased in relation to the level before the drinking test. It can be effected by corneal hydration and the water absorption by the cornea near the limbus. This is due to the different packing density of the corneal stroma. The lamellas are arranged more densely in the anterior and middle parts of the stroma than in the posterior one. In addition, they are also more hydrated in the central part, as a result of which the back part of the stroma can swell easily due to the less frequent packing of these fibres [28]. Moreover, the observation of viscoelastic behaviour in the intact human cornea may be explained by the influence of corneal hydration on the stress distribution between corneal lamellae. In the swollen cornea, only the anterior corneal lamellae are able to take up tension, whereas the posterior lamellae will be slack. Clinically, this can be observed as folds in Descement’s membrane. During the pressure-induced reduction in corneal volume, the posterior lamellae elongate and take up some of the corneal stress, and the stress on the anterior lamellae will decrease. Eliasson and Maurice [29], after studying the displacement of the cornea surfaces induced by corneal thinning, concluded that stress distribution across the corneal stroma is even in the normo-hydrated human cornea in vivo [30].

The statistical significance of CH and CRF parameters in glaucoma diagnosis have been reported previously [10,31,32,33]. However, most studies presented the comparison of ORA parameters between the healthy and glaucomatous eyes. Little work has been conducted on the postoperative diagnosis and raw data of ORA results [14,34]. The findings of the present study indicate that the analysis of ORA parameters could be utilized in postoperative and treated glaucoma diagnostics [14,35].

## Figures and Tables

**Table 1:** Demographic data.

	Control Group		XEN GelStent	
	Mean ± SD	Range	Mean ± SD	Range
**GAT** [mm Hg]	15.9 ± 3.1	10–22	14.4 ± 3.0	9–19
**CCT** [μm]	534 ± 45	436–592	531 ± 39	457–589
**ACD** [mm]	3.05 ± 0.80	1.88–4.28	3.13 ± 0.87	1.82–4.20
**AXL** [mm]	23.54 ± 1.12	23.43–26.47	23.41 ± 0.83	21.67 ± 24.88

GAT–intraocular pressure (Goldmann tonometry), CCT–central corneal thickness, ACD–anterior chamber depth, AXL–eyeball axial length.

**Table 2:** The WDT results on the intraocular pressure (IOPcc—corneal-compensated intraocular pressure, IOPg—Goldmann-correlated intraocular pressure) in the control and XEN GelStent groups.

	IOPg [mmHg]			IOPcc [mmHg]		
	Control	XEN GelStent	*p*-Value	Control	XEN GelStent	*p*-Value
Before	14.7 ± 4.4	12.8 ± 4.7	0.13 ^I^	16.5 ± 3.9	14.6 ± 4.0	0.21 ^II^
10 min	17.2 ± 3.7	14.9 ± 4.7	0.08 ^I^	19.1 ± 3.6	16.8 ± 4.3	0.13 ^I^
25 min	17.1 ± 3.1	14.8 ± 4.3	0.10 ^II^	19.1 ± 3.1	16.8 ± 4.3	0.09 ^I^
40 min	16.9 ± 4.3	13.9 ± 4.1	0.06 ^I^	19.2 ± 3.8	15.9 ± 3.9	0.05 ^I^
55 min	16.9 ± 5.5	13.9 ± 4.4	0.08 ^II^	18.9 ± 5.4	16.0 ± 4.1	0.12 ^II^
*p*-value	<0.005 ^IV^	<0.001 ^III^		<0.05 ^IV^	<0.001 ^III^	

^I^-*t*-test, ^II^-Wilcoxon test, ^III^-ANOVA test, ^IV^-Friedman test.

**Table 3:** The corneal hysteresis (CH) and corneal resistance factor (CRF) during the water drinking test (WDT) in the control and XEN GelStent groups.

	CH [mmHg]			CRF [mmHg]		
	Control	XEN GelStent	*p*-Value	Control	XEN GelStent	*p*-Value
Before	9.2 ± 1.7	9.6 ± 1.7	0.27 ^I^	9.2 ± 2.2	8.9 ± 2.3	0.35 ^I^
10 min	8.9 ± 1.9	9.1 ± 1.8	0.52 ^I^	9.6 ± 2.0	9.1 ± 2.1	0.06 ^I^
25 min	8.8 ± 1.5	9.1 ± 1.6	0.31 ^I^	9.5 ± 1.6	9.1 ± 1.7	0.11 ^I^
40 min	8.6 ± 1.7	9.2 ± 1.7	0.10 ^I^	9.3 ± 2.1	8.9 ± 1.9	0.21 ^I^
55 min	8.8 ± 1.8	9.1 ± 1.5	0.31 ^I^	9.5 ± 2.1	8.9 ± 1.8	0.11 ^I^
*p*-value	<0.05 ^III^	0.17 ^III^		0.17 ^III^	0.41 ^III^	

^I^-*t*-test, ^III^-ANOVA test.

**Table 4:** Amplitudes of the first and second applanation (respectively A1 and A2) during the water drinking test (WDT) in the control and XEN GelStent groups.

	A1 [–]			A2 [–]		
	Control	XEN GelStent	*p*-Value	Control	XEN GelStent	*p*-Value
Before	570 ± 140	540 ± 170	0.56 ^I^	430 ± 90	410 ± 100	0.52 ^I^
10 min	530 ± 150	530 ± 120	0.83 ^I^	420 ± 110	420 ± 80	0.99 ^I^
25 min	510 ± 170	480 ± 160	0.48 ^I^	380 ± 100	380 ± 100	0.99 ^I^
40 min	430 ± 150	450 ± 140	0.66 ^I^	380 ± 120	340 ± 100	<0.05 ^I^
55 min	430 ± 140	440 ± 160	0.70 ^II^	360 ± 110	360 ± 110	0.76 ^II^
*p*-value	<0.001 ^III^	<0.05 ^IV^		<0.005 ^IV^	<0.001 ^III^	

^I^-*t*-test, ^II^-Wilcoxon test, ^III^-ANOVA test, ^IV^-Friedman test.

**Table 5:** The time of the first and second applanation (respectively *t*_1_ and *t*_2_) along with the interspace between applanations (Δ*t*) during the water drinking test (WDT) in the control and XEN GelStent groups.

	*t*_1_ [ms]			*t*_2_ [ms]			Δ*t* [ms]		
	Control	XEN GelStent	*p*-Value	Control	XEN GelStent	*p*-Value	Control	XEN GelStent	*p*-Value
Before	6.65 ± 0.5	6.43 ± 0.57	0.10 ^I^	15.55 ± 0.17	15.62 ± 0.22	<0.05 ^I^	8.90 ± 0.45	9.19 ± 0.56	<0.05 ^I^
10 min	6.90 ± 0.39	6.65 ± 0.51	0.05 ^I^	15.55 ± 0.17	15.59 ± 0.19	0.29 ^I^	8.64 ± 0.34	8.94 ± 0.5	0.06 ^I^
25 min	6.9 ± 0.4	6.6 ± 0.48	<0.05 ^I^	15.52 ± 0.17	15.58 ± 0.19	0.26 ^I^	8.62 ± 0.39	8.97 ± 0.5	<0.05 ^I^
40 min	6.87 ± 0.51	6.58 ± 0.45	0.07 ^I^	15.47 ± 0.21	15.52 ± 0.21	0.38 ^I^	8.61 ± 0.56	8.94 ± 0.47	0.10 ^I^
55 min	6.86 ± 0.5	6.52 ± 0.52	0.05 ^I^	15.52 ± 0.22	15.54 ± 0.22	0.67 ^I^	8.66 ± 0.59	9.02 ± 0.5	0.10 ^I^
*p*-value	<0.005 ^II^	<0.005 ^II^		0.13 ^II^	0.05 ^II^		<0.05 ^II^	<0.005 ^II^	

^I^-*t*-test, ^II^-ANOVA test.

## Data Availability

The data will be available via online access according to the Clinical Trials registration statement.

## Supplementary Materials

The following supporting information can be downloaded at: https://www.mdpi.com/article/10.3390/jcm11112962/s1; Table S1. Demographic and clinical characteristics of the study groups (XEN group and Control group). All participants have bilateral POAG. One eye of each patient was assigned to the XEN group and the other to the Control group (the POAG group); Table S2. Variables and definitions of the corneal deformation signal parameters obtained by Ocular Response Analyzer; Table S3. Summary of parameters obtained from the corneal deformation signal parameters obtained by Ocular Response Analyzer, divided to the parameters that refers to the first and second applanation.
