# Supplementary material for: Corneal Behavior during Tonometer Measurement during the Water Drinking Test in Eyes with XEN GelStent in Comparison to Non-Implanted Eyes
Source: J Clin Med. 2022 May 24;11(11):2962. doi: 10.3390/jcm11112962 (PMC9181234; doi:10.3390/jcm11112962)
Supplement: Supplementary file 1 [file jcm-11-02962-s001.zip › jcm-1677278-supplementary.pdf]

Table S1. Demographic and clinical characteristics of the study groups (XEN group and Control group). All PARTICIPANTS have bilateral POAG. One eye of each patient was assigned to the XEN group and the other to the Control group (the POAG group).

	**XEN Group** n = 18		**Control Group** n = 18		
	**Mean ± SD**	**95% CI**	**Mean ± SD**	**95% CI**	**p-Value** *
SE [D]	–0.75 ± 1.0	-2.5–1.50	–0.25 ± 0.75	–2.5–1.75	0.59
CCT [μm]	534 ± 36	521–548	535 ± 41	516–555	0.51
ACD [μm]	3.24 ± 0.77	2.91–3.57	3.01 ± 0.72	2.56–3.46	0.57
AXL [μm]	23.9 ± 1.7	23.1–24.4	23.1 ± 1.4	22.9–24.1	0.73
BCVA [logMAR]	0.06 ± 0.10	-0.02–0.22	0.08 ± 0.12	0.00–0.20	0.81
MD [dB]	–8.8 ± 8.1	−11.7 – −6.5	–8.7 ± 8.5	−13.3 – −4.3	0.92
PSD [dB]	6.1 ± 4.1	4.9– 7.2	6.1 ± 3.9	4.1–8.1	0.74
RNFLT [μm]	62 ± 11	–	64 ± 14	–	0.87
Number of patients on topical medication	3		18		–
Number of patients on systemic medication	0		0		–

Mann-Whitney U test; CCT - central corneal thickness, ACD - anterior chamber depth, AXL - axial length of the globe, BCVA - best-corrected visual acuity, MD - mean deviation (perimetry), PSD - pattern standard deviation (perimetry), RNFLT - retinal nerve fiber layer thickness, SE-spherical equivalent

Table S2. Variables and definitions of the corneal deformation signal parameters obtained by Ocular Response Analyzer

Variable	Description
IOPg [mmHg]	Goldmann correlated intraocular pressure
IOPcc [mmHg]	Corneal compensated intraocular pressure
CH [mmHg]	Corneal hysteresis; parameter describing corneal viscosity
CRF [mmHg]	Corneal resistance factor
WS	Waveform score - quality factor informing about the reliability of the measurement
aindex, bindex	Smoothness – Degree of "non-monotonicity" of rising and falling edges of peak1 and peak2 (normalized by area)
p1area, p2area	Area of peak1 or peak2 (sum of values) derived from upper 75% of applanation peak
p1area1, p2area1	Area of peak1 or peak2 (sum of values) derived from upper 50% of applanation peak

aspect1, aspect2	Aspect ratio of peak1 or peak 2 (height/width) derived from upper 75% of applanation peak
aspect11, aspect21	Aspect ratio of peak1 or peak 2 (height/width) derived from upper 50% of applanation peak
uslope1, uslope2	Upslope of peak1 (base to peak value of peak1) or peak2 (base to peak value of peak2) derived from upper 75% of applanation peak
uslope11, uslope21	Upslope of peak1 (base to peak value of peak1) or peak2 (base to peak value of peak2) derived from upper 50% of applanation peak
dslope1, dslope2	Downslope of peak1 (base to peak value of peak1) or f peak2 (base to peak value of peak2) derived from upper 75% of applanation peak
dslope11, dslope21	Downslope of peak1 (base to peak value of peak1) or f peak2 (base to peak value of peak2) derived from upper 50% of applanation peak
w1, w2	Width of peak1 at base of peak1 region and peak 2 at base of peak2 region derived from upper 75% of applanation peak
w11, w21	Width of peak1 at base of peak1 region and peak 2 at base of peak2 region derived from upper 50% of applanation peak
h1, h2	Height of peak1 (from lowest to highest value in peak1 region) or peak2 (from lowest to highest value in peak2 region) derived from upper 75% of applanation peak
h11, h21	Height of peak1 (from lowest to highest value in peak1 region) or peak2 (from lowest to highest value in peak2 region) derived from upper 50% of applanation peak
dive1, dive2	Absolute value of monotonic decrease on downslope part of peak1 or peak2<br>starting at the peak value derived from upper 75% of applanation peak
path1, path2	Absolute value of path length around peak1 or peak2 derived from upper 75% of applanation peak
path11, path21	Absolute value of path length around peak1 or peak2 derived from upper 50% of applanation peak
mslew1, mslew2	Maximum single step increase in rise of peak1 or peak2 derived from upper 75% of applanation peak
slew1, slew2	Aspect ratio of dive1 or dive2 (value of dive divided by width of dive region) derived from upper 75% of applanation peak
aplhf	High-frequency noise in region between peaks (normalized by product of average of peak heights times width of region)

The 2nd applanation region is time reversed so that the upslope (uslope notation) of peak2 is actually a downslope (dslope notation) in real time.

Table S3. Summary of parameters obtained from the corneal deformation signal parameters obtained by Ocular Response Analyzer, divided to the parameters that refers to the first and second applanation.

		Before	10 min	25 min	40 min	55 min	p-value
**First applanation (peak1)**							

**aindex**	Control	8.4 ± 0.9	8.8 ± 1.2	8.3 ± 1.5	8.2 ± 1.2	8.7 ± 1.3	0.78 IV
	XEN-Gel	8.7 ± 1.3	8.8 ± 0.7	8.2 ± 1.1	8.8 ± 0.6	8.8 ± 0.9	0.37 IV
**p1area**	Control	4200 ± 1300	3800 ± 1100	3800 ± 1400	3340 ± 990	3100 ± 1100	<0.001 III
	XEN-Gel	4200 ± 1500	3900 ± 1000	3800 ± 1100	3400 ± 1000	3400 ± 1100	<0.05 IV
**p1area1**	Control	1800 ± 630	1620 ± 510	1640 ± 690	1370 ± 460	1350 ± 510	<0.001 III
	XEN-Gel	1800 ± 790	1660 ± 510	1580 ± 540	1450 ± 460	1420 ± 490	<0.05 III
**aspect1**	Control	18.4 ± 4.6	16.9 ± 5.6	15.3 ± 5.4	13.6 ± 5.1	12.8 ± 5.4	<0.001 III
	XEN-Gel	17.4 ± 6.2	16.7 ± 4.7	15.2 ± 5.4	13.8 ± 6.0	13.4 ± 6.2	<0.05 III
**aspect11**	Control	24.5 ± 7.5	23 ± 9.6	20.6 ± 6.3	18.3 ± 6.2	17 ± 7.5	<0.005 III
	XEN-Gel	22.5 ± 8.4	21.9 ± 7.1	20.3 ± 7.9	18.5 ± 9.8	17 ± 8.5	<0.05 IV
**h1**	Control	420 ± 100	390 ± 110	370 ± 120	320 ± 100	300 ± 110	<0.001 III
	XEN-Gel	400 ± 130	382 ± 93	360 ± 110	330 ± 110	320 ± 120	<0.01 III
**h11**	Control	279 ± 69	257 ± 75	245 ± 83	217 ± 66	202 ± 74	<0.001 III
	XEN-Gel	268 ± 85	255 ± 62	242 ± 73	218 ± 77	211 ± 80	<0.01 III
**w1**	Control	23.3 ± 2.1	23.6 ± 2.2	24.8 ± 2.5	25.6 ± 4.4	25.3 ± 4.3	**0.13 IV**
	XEN-Gel	24.4 ± 3.3	23.7 ± 2.2	24.8 ± 2.8	25 ± 2.9	25.1 ± 3.5	**<0.05 IV**
**w11**	Control	12.2 ± 2	12.6 ± 2.2	12.9 ± 2.4	13.5 ± 3.3	13.7 ± 4.1	0.72 IV
	XEN-Gel	13.2 ± 2.7	12.6 ± 1.9	13 ± 2.7	13.3 ± 2.9	13.8 ± 2.8	0.47 III
**uslope1**	Control	71 ± 21	65 ± 23	61 ± 17	56 ± 19	48 ± 21	<0.001 III
	XEN-Gel	68 ± 21	62 ± 18	58 ± 20	56 ± 29	51 ± 27	<0.05 IV
**uslope11**	Control	65 ± 20	62 ± 27	56 ± 17	55 ± 22	46 ± 21	<0.05 III
	XEN-Gel	62 ± 17	58 ± 16	57 ± 22	51 ± 26	49 ± 23	<0.05 IV
**dslope1**	Control	26 ± 7	24 ± 8	21 ± 8	19 ± 8	18 ± 8	<0.001 III
	XEN-Gel	25 ± 9	24 ± 8	22 ± 8	19 ± 8	19 ± 9	<0.05 IV
**dslope11**	Control	41 ± 13	38 ± 17	33 ± 12	29 ± 11	28 ± 13	<0.005 III
	XEN-Gel	37 ± 15	37 ± 15	34 ± 16	29 ± 15	27 ± 13	<0.05 III
**dive1**	Control	320 ± 100	320 ± 110	310 ± 120	266 ± 92	250 ± 120	<0.05 III
	XEN-Gel	340 ± 120	321 ± 78	320 ± 110	270 ± 110	270 ± 120	<0.05 III
**path1**	Control	21.7 ± 3.9	21 ± 3.8	20.6 ± 3.5	20.7 ± 3.8	20.4 ± 6.3	0.71 IV
	XEN-Gel	20.1 ± 2.8	20.2 ± 2.8	20.5 ± 3.9	19.3 ± 3.3	19.3 ± 3.4	0.35 III
**path11**	Control	31.5 ± 5.4	32 ± 8	30.4 ± 7.8	31.9 ± 7.2	30.1 ± 10.1	0.58 IV
	XEN-Gel	30.2 ± 5.5	30.2 ± 6.2	31.3 ± 9.7	28.1 ± 4.4	29.2 ± 7.6	0.43 IV
**mslew1**	Control	119 ± 33	108 ± 33	103 ± 32	93 ± 30	82 ± 33	<0.01 IV
	XEN-Gel	116 ± 37	107 ± 33	101 ± 33	92 ± 40	87 ± 41	<0.05 IV
**slew1**	Control	69 ± 20	67 ± 26	63 ± 17	57 ± 20	49 ± 24	**<0.01 III**
	XEN-Gel	69 ± 21	63 ± 19	62 ± 20	57 ± 29	51 ± 28	**0.05 IV**
**Second applanation (peak2)**							
**bindex**	Control	8.7 ± 1.3	8.6 ± 1.1	8.2 ± 1.6	8.3 ± 1.5	9.0 ± 0.9	0.18 IV
	XEN-Gel	8.9 ± 1.2	8.8 ± 0.8	8.4 ± 1.3	8.4 ± 1.4	8.7 ± 1.3	0.27 IV

**p2area**	Control	2480 ± 740	2350 ± 670	2280 ± 590	2390 ± 700	2210 ± 680	0.06 III
	XEN-Gel	2250 ± 650	2400 ± 710	2290 ± 600	2200 ± 730	2340 ± 760	0.39 III
**p2area1**	Control	1050 ± 370	980 ± 290	940 ± 270	1000 ± 310	950 ± 320	0.09 III
	XEN-Gel	950 ± 280	1010 ± 320	940 ± 260	910 ± 310	990 ± 350	0.33 III
**aspect2**	Control	17.5 ± 5	16.7 ± 6.1	14.7 ± 6.4	14.9 ± 6.2	13.6 ± 6.2	<0.05 III
	XEN-Gel	19.4 ± 6.4	17.6 ± 4.9	15.3 ± 5.2	13.4 ± 5.8	14.1 ± 7.0	<0.001 III
**aspect21**	Control	25.6 ± 8.6	24.3 ± 9	22.8 ± 10.2	21.2 ± 8.6	18.4 ± 8.6	<0.05 IV
	XEN-Gel	28.5 ± 9.9	25 ± 7.5	22.3 ± 8.7	19.4 ± 10.1	19.8 ± 11.9	<0.005 IV
**h2**	Control	319 ± 66	304 ± 91	286 ± 78	283 ± 77	259 ± 88	<0.01 III
	XEN-Gel	316 ± 78	310 ± 63	285 ± 70	254 ± 73	268 ± 89	<0.01 III
**h21**	Control	213 ± 44	203 ± 61	191 ± 52	189 ± 51	173 ± 58	<0.01 III
	XEN-Gel	210 ± 52	207 ± 42	190 ± 47	169 ± 49	178 ± 60	<0.005 III
**w2**	Control	20.1 ± 3.3	20.3 ± 3.4	21.5 ± 4.1	21.8 ± 4.8	21.7 ± 4.5	**0.19 III**
	XEN-Gel	19.2 ± 4	19.6 ± 3.9	21.5 ± 4.9	22 ± 5.2	22 ± 5.5	**<0.01 IV**
**w21**	Control	9.6 ± 2	9.8 ± 2.3	10.4 ± 2.4	10.8 ± 3.3	11.3 ± 3.2	**0.18 IV**
	XEN-Gel	9.0 ± 2.0	9.5 ± 2.4	10.5 ± 2.9	11.0 ± 3.1	11.4 ± 3.7	**<0.005 III**
**uslope2**	Control	82 ± 23	82 ± 30	81 ± 33	70 ± 24	65 ± 30	<0.05 III
	XEN-Gel	92 ± 27	89 ± 26	78 ± 27	60 ± 24	64 ± 28	<0.001 III
**uslope21**	Control	41 ± 16	38 ± 14	33 ± 16	33 ± 15	28 ± 15	**0.13 III**
	XEN-Gel	73 ± 28	72 ± 24	64 ± 24	47 ± 18	51 ± 19	**<0.001 III**
**dslope2**	Control	23 ± 7	22 ± 8	19 ± 9	19 ± 8	17 ± 8	<0.05 III
	XEN-Gel	26 ± 10	23 ± 7	20 ± 6	17 ± 8	19 ± 9	<0.05 III
**dslope21**	Control	68 ± 16	66 ± 27	67 ± 28	61 ± 21	54 ± 25	<0.05 IV
	XEN-Gel	45 ± 20	37 ± 11	34 ± 15	30 ± 18	31 ± 22	<0.05 IV
**dive2**	Control	235 ± 68	236 ± 86	222 ± 73	217 ± 66	198 ± 70	**0.10 IV**
	XEN-Gel	243 ± 93	242 ± 69	208 ± 55	188 ± 66	204 ± 70	**<0.05 III**
**path2**	Control	25.3 ± 4.8	25 ± 4.2	25.1 ± 5.2	23.4 ± 4.5	22.2 ± 5.1	<0.05 III
	XEN-Gel	27.5 ± 6.3	25.8 ± 4.8	24.6 ± 5.3	23.2 ± 4.8	22.9 ± 5.8	<0.005 IV
**path21**	Control	36.7 ± 6.6	35.4 ± 5.6	37 ± 7.1	35.1 ± 8.2	32.2 ± 7.5	**0.07 IV**
	XEN-Gel	37.2 ± 8.3	35.7 ± 5.5	35.8 ± 8.1	33.1 ± 6.8	32 ± 7.8	**<0.05 III**
**mslew2**	Control	133 ± 30	130 ± 39	121 ± 38	114 ± 38	106 ± 40	<0.01 III
	XEN-Gel	137 ± 27	137 ± 28	122 ± 36	102 ± 32	107 ± 45	<0.001 IV
**slew2**	Control	82 ± 22	84 ± 29	83 ± 32	72 ± 23	65 ± 29	<0.05 III
	XEN-Gel	92 ± 27	90 ± 27	81 ± 28	61 ± 23	65 ± 27	<0.001 III
